# Turning of Additively Manufactured Ti6Al4V: Effect of the Highly Oriented Microstructure on the Surface Integrity

**DOI:** 10.3390/ma14112842

**Published:** 2021-05-26

**Authors:** Lucia Lizzul, Rachele Bertolini, Andrea Ghiotti, Stefania Bruschi

**Affiliations:** Department of Industrial Engineering, University of Padova, Via Venezia 1, 35131 Padova, Italy; lucia.lizzul@phd.unipd.it (L.L.); rachele.bertolini@unipd.it (R.B.); andrea.ghiotti@unipd.it (A.G.)

**Keywords:** titanium, additive manufacturing, turning, surface integrity

## Abstract

Additive manufacturing processes induce a high orientation in the microstructure of the printed part due to the strong thermal gradients developed during the process caused by the highly concentrated heat source that is used to melt the metal powder layer-by-layer. The resulting microstructural anisotropy may have an effect on the post-processing operations such as machining ones. This paper investigates the influence of the anisotropy in turning operations carried out on laser powder bed fused Ti6Al4V parts manufactured with different scanning strategies. The machinability under both transverse and cylindrical turning operations was assessed in terms of surface integrity, considering both surface and sub-surface aspects. The effect of the different cooling conditions, that is flood and cryogenic ones, was studied as well. The outcomes showed that the microstructural anisotropy had a remarkable effect on the machining operations and that the cryogenic cooling enhanced the effect of the anisotropy in determining the surface integrity.

## 1. Introduction

At the industrial level, the Additive Manufacturing (AM) of metals has been developing to meet the demands of process automation and for the reduction of material wastage [1,2]. AM parts are, in fact, manufactured by deposing and subsequently melting material layer-by-layer, starting from a digital model that helps in accommodating complex design together with the ease of model and data manipulation. However, a thorough understanding of the AM parts mechanical properties has yet to be achieved because of the peculiar microstructure deriving by the AM process itself, making it difficult to integrate this technology into the process chain. Strong thermal gradients develop during the deposition process due to the concentrated heat source used to melt the metal powder, such as laser or electron beams, inducing a highly oriented microstructure along the Build-up Direction (BD) of the part. The resulting anisotropy depends principally on the BD of the part, but it is strictly related also to other factors, such as the sample height, dimensions, and support structure, as well as the AM process parameters [3,4], regardless of the post-AM heat treatment [5,6].

The AM-induced microstructural features are known to affect both the part performances, such as fatigue behavior or corrosion resistance [7,8] and recent studies demonstrated their effect also on the machinability of such parts [9]. In fact, machining operations are usually required to achieve the final geometrical tolerances and surface finish as well as improve in-service performances of the AM parts [10]. Among the AM metal alloys, Ti6Al4V titanium alloy is one of the most utilized for the production of high-value parts in both the aerospace and biomedical fields [11]. Compared to the conventionally manufactured parts, the use of AM indeed allows for the reduction of the mass of aircraft components, leading to lighter structures together with reduced costs, material wastage, and lead times [10]. On the other hand, for the medical sector AM allows the production of custom-shaped implants as well as porous surface features that promote osseointegration [12]. However, the titanium alloys fall into the category of the so-called difficult-to-cut metals, because of their high strength and chemical reactivity to oxygen, together with low thermal conductivity, which leads to high temperatures in the cutting zone, affecting the part surface and sub-surface characteristics, which, in turn, strongly impact the part functional performances [13,14]. When compared to traditional manufacturing processed parts, the AM ones usually have higher mechanical properties that further negatively affect their machinability [11]. For this reason, in recent years, the issue of machining AM titanium alloys has grown in importance.

The influence on the tool wear and surface topography in the milling of Laser Powder Bed Fused (LPBFed) Ti6Al4V parts at different growth inclination angles was investigated in [15]. The tool wear, as well as the surface roughness, was found to decrease with the decreasing of the inclination angle because of the influence of weak discontinuity zones along the BD that helped the material removal when favorably oriented compared to the cutting edge of the cutting tool. There is still a lack of studies concerning the AM-induced anisotropy effect in the turning of AM Ti6Al4V components. Some studies were carried out to compare the surface integrity and tool wear of AM Ti6Al4V with the same wrought alloy [16,17], showing the worst machinability of the former. As shown in the above-mentioned published works, even if some researchers have studied the machinability of AM parts, none of them have considered AM-induced anisotropy in relation to turning operations. Therefore, this paper is dedicated to an in-depth study of the machinability in terms of surface integrity of Ti6Al4V AM samples when changing cutting directions in turning operations. First, Ti6Al4V samples were LPBFed by adopting two different scanning strategies, namely chessboard and stripes. Afterward, the samples were machined under different cooling conditions, including flood and cryogenic cooling, as well as different turning operations, namely cylindrical turning and facing. The microstructure, nanohardness, surface roughness and defects of the machined samples were characterized. Finally, the relationship between the material anisotropy and process condition was assessed.

## 2. Materials and Methods

### 2.1. Material and Processing Parameters

LPBFed Ti6Al4V (grade 5) samples, with nominal chemical composition reported in Table 1, were manufactured in the laboratory in form of 25 mm diameter cylindrical bars with 60 mm height. The EOSINT™ M 280 3D printer (by EOS GmbH, Krailling, Germany) was used for the parts manufacturing, which is characterized by a building volume of 250 × 250 × 325 mm^3^. The heating source is a ytterbium fiber laser with a maximum power of 400 W and a focus diameter of 100 µm.

To manufacture the samples the following processing parameters were utilized: 280 W laser power, 1200 mm/s scanning speed, 100 µm hatch spacing, and 30 µm layer thickness. The scanning strategy was varied, in particular, the stripes and the chessboard strategies were employed. In the first case, each layer was composed of 5 mm wide stripes with a scan vector that was rotated by 67° for each successive layer. In the chessboard scanning strategy, the built area was divided into 5 × 5 mm^2^ blocks perpendicular to each other with an overlapping of 0.04 mm. Each layer was shifted by 2.5 mm in both the X and Y directions of the scan area.

The LPBFed bars underwent a post-processing heat treatment to relieve the thermal stresses, stabilize the microstructure, and reduce the porosities for an overall improvement of the mechanical properties [18,19]. The heat treatment was executed in an inert argon atmosphere with a heating rate of approximately 6 °C/min up to 950 °C held for 30 min followed by furnace cooling using the RHF™ laboratory furnace (by Carbolite Gero Ltd., Sheffield, UK). The density of the LPBFed samples was then calculated by applying the Archimedes’ method. LPBFed bars with a density greater than 99.9% ± 0.2% were obtained even before the heat treatment, indicating fully dense parts.

**Table 1:** Chemical composition of the Ti6Al4V powder (weight %), according to ASTM F2924-14 [20].

Al	V	Fe	O	C	N	H	Ti
5.50–6.75	3.5–4.5	<0.30	<0.20	<0.08	<0.05	<0.015	Bal.

### 2.2. Machining Tests

Machining tests were carried out on a Mori Seiki^TM^ lathe (by DMG Mori, Bielefeld, Germany) located at lab TE.SI. in Rovigo. Turning operations were performed by adopting a PVD TiAlN coated CNMG120404-SM 1105 insert (by Sandvik Coromant, Sandviken, Sweden) characterized by a rake angle (α) of 7° and corner radius (R) of 0.397 mm.

The machining trials comprised two steps: first, the bar was roughened in order to achieve a diameter of 24 mm by using a depth of cut (*ap*) of 0.5 mm, feed (*f*) of 0.2 mm/rev, and cutting speed (*V*) of 80 m/min. Afterward, in the case of cylindrical turning, a single finishing pass was performed to reach a final diameter of 23.7 mm by using an *ap* of 0.15 mm and *f* of 0.15 mm/rev. On the contrary, in the case of facing, a single finishing pass was performed to reduce the bar length of 0.3 mm by using an *f* of 0.15 mm and *ap* of 0.15 mm/rev. Finally, for both turning configurations, 5 mm thick cylindrical samples were cut for subsequent investigations.

**Figure 1:**
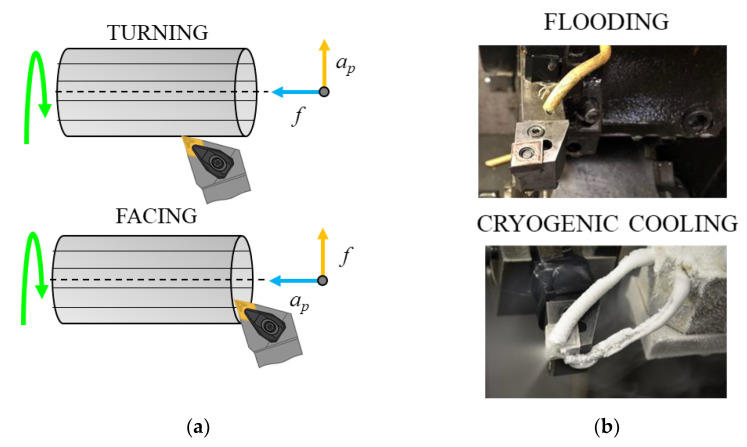
Scheme of the cylindrical turning and facing operations: (**a**) flooding and cryogenic cooling experimental setup (**b**).

In cylindrical turning the tool moves parallel to the rotation axis of the workpiece, whereas, during facing, it moves orthogonally to its axis, as schematically depicted in Figure 1a. This makes possible a different interaction between the cutting tool and the microstructure anisotropy; indeed, the tool travels parallel and orthogonal to the grain growth direction (graphically depicted as black lines within the bar in Figure 1a) when turning and facing, respectively.

The machining tests were carried out under both conventional flood and cryogenic conditions. The first machining strategy is mandatory to machine difficult-to-cut metals, like titanium alloys, in order to limit temperature rises during cutting. Cryogenic machining is chosen as an alternative approach since it is an emerging alternative cooling condition capable to limit tool wear and increase surface integrity of the machined workpiece. Flooding refers to the spray of semi-synthetic cutting fluid Monroe™ Astro-Cut HD XBP (by Monroe Fluid, Hilton, NY, USA) mixed with water (1:20 mixing ratio) using a single nozzle directed to the cutting tool. Cryogenic cooling refers to the spray of liquid nitrogen (LN_2_) simultaneously to the flank and rake faces of the tool by means of two copper nozzles of 0.9 of diameter. The cryogenic cooling apparatus includes a dewar, in which the liquid nitrogen is stored at 15 bar, a vacuum pipe used to carry LN_2_, and a specifically designed setup to deliver LN_2_ to the cutting zone at a nominal temperature of −192 °C. Thorough information on the utilized cryogenic setup is reported in a previous study [21]. In the present experimental condition, the mass rate was set to 0.058 kg/s, while the cooling capacity to 24.7 kW. 

Both turning operations and cooling conditions were applied to samples obtained with different scanning strategy. A new cutting insert was used for each specimen so to avoid any influence of the tool wear on the experimental results and to isolate the effect of the microstructural anisotropy on the surface integrity results.

The whole experimental plan is summarized in Table 2. 

### 2.3. Characterization of the Machined Samples

The LPBFed Ti6Al4V samples were prepared for the metallographic inspections by carefully sectioning the bars at the same height, followed by hot mounting. The sections were ground using up to 4000 grit SiC abrasive papers and polished with a 1:4 mix of colloidal silica suspension and hydrogen peroxide 30% on a polyester cloth. After 15 s of etching with the Kroll’s reagent, the samples were cleaned with warm tap water and immediately dried with an air blast. Since the two machining operations had different cutting directions, two cross-sections were always examined in the metallographic analyses. The effect of cylindrical turning was evaluated on the cross-section perpendicular to the BD of the cylindrical bars, while the effect of facing was evaluated on cross-sections parallel to the BD at a distance of 2 mm from the external radius of the bars.

Using a high-definition digital camera of the DMRE™ optical microscope (by Leica, Wetzlar, Germany), micrographs were taken at 50× and 1000× magnification for microstructural analysis. The β grains widths were measured on six separate images using the 9.5 version of Matlab™ software following the ASTM E112 line intersection method [22]. To measure the extent of the plastic deformation of the lamellae induced by the machining operations, referred to as the Severe Plastic Deformation (SPD) surface layer, the ImageJ™ software was utilized on ten different micrographs.

Vickers microhardness was measured with the Durimet™ tester (by Leitz, Wetzlar, Germany) with a load of 50 gf for 15 s dwelling time according to the ASTM E92-17 standard [23]. For each specimen, bulk microhardness was assessed with thirty indentations across the mounted cross-sections. The subsurface strain hardening induced by machining was measured using a diamond Berkovich tip of the iMicro™ nanoindenter (by Nanomechanics Inc, Oak Ridge, TN, USA). All the indentations were performed using a load of 30 mN up to 20 μm below the machined surface. For reliable measurement, three 4 × 5 matrices of indentations were performed on each specimen. Rows and columns were spaced 5 μm to each other. The bulk nanohardness was measured as well, performing three 4 × 4 matrices sufficiently distant from the surface. All of the above measurements were collected in order to determine the average values and standard deviations.

To analyze the presence of defects and micro-morphology of the machined surfaces, the FEI QUANTA™ 450 (by FEI Company, Hillsboro, OR, USA) Scanning Electron Microscope (SEM) with the Backscattered Electron Detector (BSED) was utilized.

The machined surface topography was investigated using the PLU-Neox™ optical profiler (by Sensofar, Barcelona, Spain) equipped with a 20× confocal objective (by Nikon™, Tokyo, Japan) with 0.45 of numerical aperture. For each surface, three scans of 0.7 × 3.0 mm^2^ were acquired and the most relevant areal surface parameters to characterize a machined surface were analyzed and processed as defined in the ISO 25178 standards [24,25]. The parameters taken into consideration were the arithmetical mean height (*Sa*), the skewness (*Ssk*), the reduced peak height (*Spk*), and the reduced valley depth (*Svk*). The *Sa* parameter represents an overall measure of the texture comprising the surface generally utilized to describe the surface roughness of machined components. *Ssk* refers to the symmetry of the profile about the mean line and is useful to describe the load-carrying ability of a surface and its corrosion tendency. The *Ssk* is zero for an ideal normal distribution of the heights, a positive *Ssk* value indicates a majority of peaks on a surface, whereas a negative *Ssk* value designates a predominance of valleys. *Spk* is a functional parameter that represents the mean height of peaks above the core roughness that are surface sites prone to corrosion and material which is likely to be removed in rubbing surfaces. The other functional parameter is *Svk* that denotes the valley depth below the core roughness, useful to discriminate the presence of possible stress concentration sites or dales where a substance (a lubricant or a corrosive fluid) can accumulate. The average values of the areal surface parameters were reported with the relative standard deviation.

## 3. Results and Discussion

### 3.1. Microstructure of the As-Built and Heat-Treated LPBFed Samples

**Figure 2:**
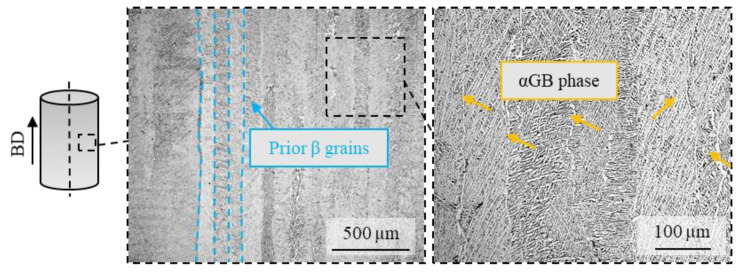
LPBFed Ti6Al4V microstructure at different magnifications (BD stands for building direction).

Figure 2 shows the microstructure along the BD of the heat-treated LPBFed Ti6Al4V printed with the chessboard strategy. In general, the microstructure is composed of three different features: (i) elongated prior β grains developed along the BD, (ii) continuous lines of αGB phase layers that decorate the β grains boundaries, and (iii) α + β lamellae within β grains organized in the Widmanstätten morphology. Thus, the microstructure of the Ti6Al4V samples is a combination of coarse columnar and fine lamellar structures, which can be ascribed to the high cooling rate (R) typical of the LPBF process, to the formation of metastable phases, and to large temperature gradients. In particular, the coarse grains are located near the boundaries of the melt pools, namely where the scan tracks overlap; these areas are melted twice, which promotes grain growth. Away from the overlaps, the grains become finer.

Changing the scanning strategy has an impact on the mean grain size: in the case of the chessboard strategy, a 32% decrease with respect to the stripes one was shown (see data reported in Table 3). It is acknowledged that the scanning strategy strongly influences both the melt pool morphology and the overlap between the layers [9]. In fact, the size of the molten pool becomes larger when the long scanning strategy is adopted. In [26], it was demonstrated that the width of the molten pool increased from 172 µm to 190 µm at fixed laser power when the scanning strategy shifted from chessboard to stripe.

Table 1 reports also the micro-hardness variations along the BD for the investigated samples. By adopting chessboard as scanning strategy, the micro-hardness increased from 307 ± 14 to 359 ± 18 HV0.05 (17% increase). This strengthening can be related to the grain refinement, ruled by the Hall–Petch relationship [27]. 

### 3.2. Microstructure of the Machined LPBFed Samples

**Figure 3:**
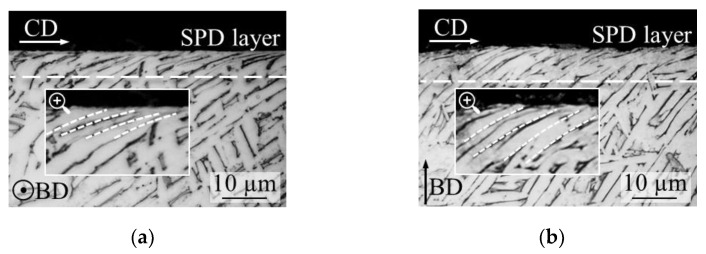
LPBFed Ti6Al4V microstructure of the sample scanned with the stripes strategy and machined under cryogenic condition: cylindrical turning (**a**) and facing (**b**).

The microstructures of the LPBFed Ti6Al4V samples after cylindrical turning and facing under cryogenic condition are presented in Figure 3. In general, as a result of the cutting, an SPD layer below the surface towards the cutting direction (CD) was formed. This zone is solely characterized by the presence of one-directional grain boundaries. The SPD layer with decreasing gradient decreased, until, at a certain depth, no deformation of the bulk material was visible anymore.

**Figure 4:**
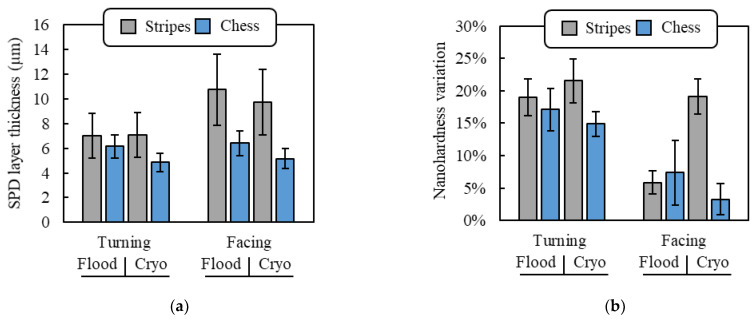
SPD layer thickness (**a**) and nanohardness of the machined sub-surface (**b**).

Figure 4a reports the thickness of the SPD layer at varying cutting conditions, whereas Figure 4b the nanohardness in correspondence of the machined surface. In general, samples obtained by facing showed a thicker SPD layer, especially for the specimen scanned with the stripes strategy. For those samples, a 68% and 89% increase in the SPD layer thickness was obtained compared to the corresponding samples manufactured under flood and cryogenic environment, respectively. This difference was reduced in the case of cylindrical turning to 14% and 45%, respectively.

The cooling effect did not have a strong impact on the SPD layer extension, being the values for samples machined under flood and cryogenic environment comparable. 

Finally, the scanning strategy had a substantial effect solely in the case of face turning. To explain the influence of the scanning strategy and cutting direction on the machined sub-surface alterations, the αGBs density must be taken into consideration. As discussed in Section 3.1, the microstructure of the LPBFed samples was characterized by the presence of αGBs along the β grains. These microstructural features typical of AMed alloys are known to affect the mechanical properties, behaving as preferential fracture paths [4,28]. The αGBs are, thus, material discontinuities that may favor the material removal during cutting as found in previous studies [15]. Being the prior β grains width lower when using the chessboard scanning strategy, the density of the weak αGBs was higher in these samples, which may have made easier the cutting operation, reducing the strength offered by the material. As already stated, differences in sub-surface alterations were particularly evident in facing operations. In fact, in such operations, the insert cuts progressively the whole cross-section of the rotating bar along its radius. This ensures that the cutting edge encounters the weak αGBs, which are along the BD, more frequently than it happens during cylindrical turning, enhancing their effect on the cutting operation. In addition, the softer the material the higher the tendency of being plastically deformed by the tool. On the contrary, in cylindrical turning, the cutting tool moves parallel to the αGB phase layers, making their presence negligible in the cutting process.

### 3.3. Surface Texture of the Machined LPBFed Samples

**Figure 5:**
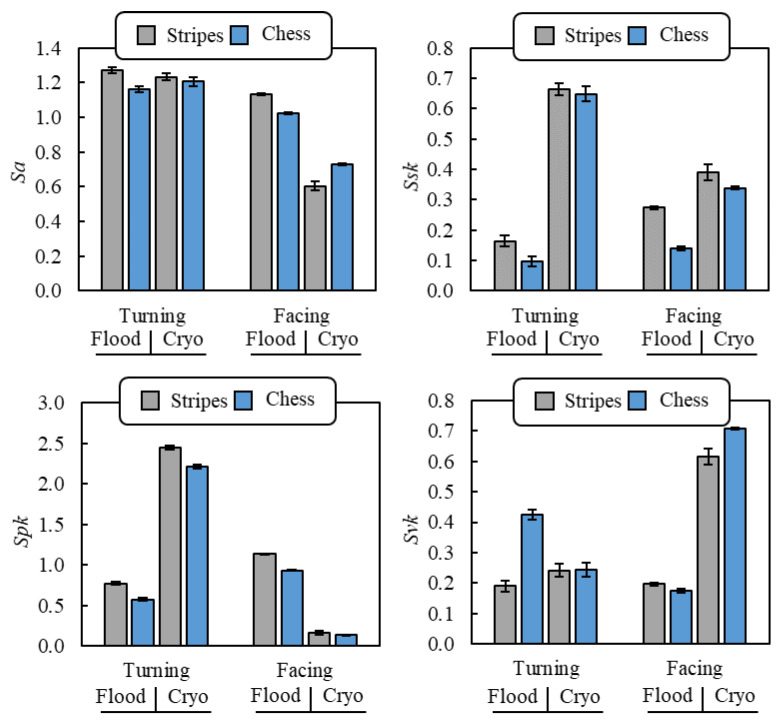
Surface roughness parameters of the machined surfaces.

Figure 5 reports the areal roughness parameters values for all the machined surfaces. Examining the parameters trend, the cutting direction had a great effect in determining the surface texture. In particular, all the examined parameters, on average, decreased between 27% and 61% in facing rather than in cylindrical turning, except for the *Svk* parameter, which increased by 55%. The same trends were found analyzing the scanning strategy influence, even if less pronounced. Specifically, the chessboard strategy led to a reduction of *Sa*, *Spk,* and *Svk* values between 3% and 18%, and to a 25% increase of the *Svk* values with respect to the stripes strategy. These results indicate that both facing and the chessboard scanning strategy allowed for the formation of machined surfaces characterized by lower roughness, lower peaks height, and principally dominated by deep valleys rather than peaks.

To understand the influence of the scanning strategy and cutting direction on the surface topography, the αGBs density must be considered, once again. As discussed in Section 3.2, being the prior β grains width lower when using the chessboard scanning strategy, the density of the weak αGBs was higher in these samples, which may have reduced the cutting forces, and, in turn, the surface roughness [29] as actually occurred. As previously stated, the αGBs effect was especially noticeable in facing operations, since the cutting edge encounters the weak αGBs more often than it does during cylindrical turning, as if it were in contact with a material with lower shear strength. This suggests why the *Sa* values measured in facing operations were lower than those measured in cylindrical turning ones, as well as why the *Sa* values found in facing were more sensitive to the different scanning strategy.

**Figure 6:**
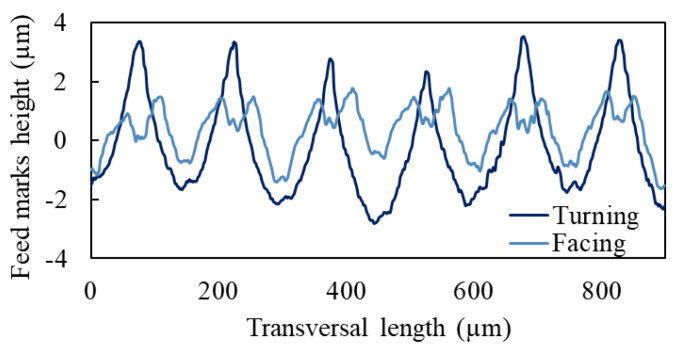
Roughness profile of the feed marks in cylindrical turning and facing under cryogenic cooling.

For what concerns the cooling condition effect, the cryogenic cooling reduced the surface roughness *Sa* by 18% with respect to the flood condition, while it had the opposite effect on the other areal parameters, with a great increase especially of the *Ssk* parameter (+202%). Therefore, cryogenic cutting led to machined surfaces dominated by high peaks and great valleys, even if overall the roughness reduced. When considering the roughness profile of the feed marks, it was noticed that facing led to the formation of a double peak, while cylindrical turning led to neat peaks, as showed in Figure 6. Both the peaks were more pronounced under cryogenic cooling condition with respect to the flood one. In the first case, this corresponded to reduced *Spk* and increased *Svk* values, with intermediate *Spk* values. In the case of cylindrical turning, the spiked peaks resulted in greater *Spk* and *Ssk* values, being the majority of the material located below the core roughness. This finding can be attributed to the lower cutting temperatures of the cryogenic condition, which hindered the local softening of the material peaks, confirming what was found in previous works on cryogenic machining [30,31]. The effect of the cryogenic cooling condition on the surface topography was especially observed between comparing the textures of the two cutting directions. In fact, when flood conditions were applied, the differences in the roughness parameters among the samples were lower. Probably the lower cutting temperatures avoided the plastic deformation of the workpiece while cutting, increasing the impact on cutting dynamics of the αGBs, as previously mentioned.

### 3.4. Surface Defects of the Machined LPBFed Samples

**Figure 7:**
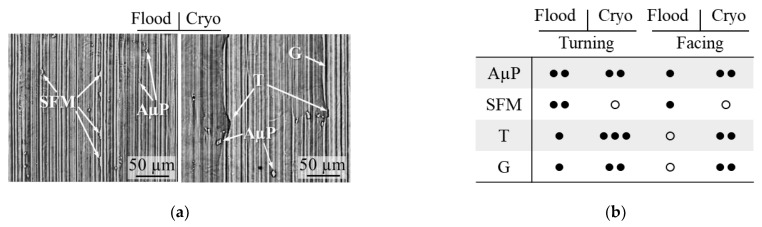
SEM images of the machined surface defects at varying cooling condition in case of cylindrical turning (**a**). The table (**b**) reports the summary of the defect entity charts (AµPM: adhered microparticles; SFM: smeared feed marks; T: tearing; G: grooves).

The SEM analyses of the machined surfaces revealed the presence of defects usually found on difficult-to-cut alloys surfaces [32]. Besides the differences in surface topography between the samples obtained with different scanning strategies, no detectable differences were found in the SEM analyses of the machined surface defects. On the contrary, when considering the effect of the cutting direction, a higher incidence of smeared feed marks and adhered microparticles were observed in cylindrical turning, which usually occurs at increasing cutting temperature. These observations can be explained by the fact that material removal in facing is favored by the presence of αGBs that may have hindered excessive heat generation in the cutting zone. The major effect was given by the cooling condition, which led to the formation of greater matrix tearing and surface grooves as showed in Figure 7. This is in accordance with the lower cutting temperatures characterizing this operation [33].

## 4. Conclusions

The impact on the machined surface integrity of the anisotropic microstructure of the AM Ti6Al4V alloy was investigated in this study. Cylindrical bars were manufactured using the LPBF technology varying the scanning strategy (stripes and chessboard). Subsequently, the bars were heat treated before being subjected to cylindrical turning and facing operations under both flood and cryogenic conditions. The main findings are:

- The scanning strategy had an impact on the size of the columnar grains (+44% grains width in the case of stripes with respect to chessboard) which is related to the material microhardness (−17% HV0.05 in the case of stripes with respect to chessboard) and the density of the αGB layers after heat treatment.
- The microstructural anisotropy deriving from the AM process greatly influenced the surface integrity of the machined samples both in terms of sub-surface (−35% SPD layer extent with −36% nanohardness in the case of stripes with respect to chessboard) and superficial features (overall stripes scanning strategy led to rougher and more spiky surfaces with respect to chessboard one).
- The weak αGBs promoted the material removal, especially when favorably oriented with respect to the cutting edge, allowing for a 28% reduction of the surface roughness and 61% reduction of peaks heights in the case of facing rather than cylindrical turning.
- Cryogenic cooling emphasized the effect of the material anisotropy in determining the surface integrity.

The research findings on the surface quality and machinability of AMed Ti6Al4V samples can provide useful guidance for their future applications in industry. This paper showed that the peculiar microstructure induced by the AM process leads to different material machinability at varying cutting direction and/or scanning strategy, presenting the best machining performances when cutting the sample surface perpendicular to the build-up direction.

## Figures and Tables

**Table 2:** Experimental plan for the machining tests.

Sample ID	Scanning Strategy	Turning Operation	Cooling Condition
S1	Stripes	Cylindrical	Flood
S2	Stripes	Cylindrical	Cryogenic
S3	Stripes	Face	Flood
S4	Stripes	Face	Cryogenic
S5	Chess	Cylindrical	Flood
S6	Chess	Cylindrical	Cryogenic
S7	Chess	Face	Flood
S8	Chess	Face	Cryogenic

**Table 3:** Mean grain size and micro-hardness of the LPBFed Ti6Al4V samples.

Scanning Strategy	Mean Size of β Grain (μm)	Hardness HV0.05
Stripes	157 ± 37	307 ± 14
Chess	106 ± 11	359 ± 18

## Data Availability

Data sharing is not applicable.
